# Prediction-Related Frontal-Temporal Network for Omission Mismatch Activity in the Macaque Monkey

**DOI:** 10.3389/fpsyt.2022.557954

**Published:** 2022-04-26

**Authors:** Yuki Suda, Mariko Tada, Takeshi Matsuo, Keisuke Kawasaki, Takeshi Saigusa, Maho Ishida, Tetsuo Mitsui, Hironori Kumano, Kenji Kirihara, Takafumi Suzuki, Kenji Matsumoto, Isao Hasegawa, Kiyoto Kasai, Takanori Uka

**Affiliations:** ^1^Department of Integrative Physiology, Graduate School of Medicine, University of Yamanashi, Chuo, Japan; ^2^Brain Science Institute, Tamagawa University, Machida, Japan; ^3^Department of Neuropsychiatry, Graduate School of Medicine, The University of Tokyo, Bunkyo, Japan; ^4^International Research Center for Neurointelligence (WPI-IRCN), The University of Tokyo Institutes for Advanced Study (UTIAS), The University of Tokyo, Bunkyo, Japan; ^5^Department of Neurosurgery, Tokyo Metropolitan Neurological Hospital, Fuchu, Japan; ^6^Department of Physiology, Niigata University School of Medicine, Chuo, Japan; ^7^Department of Social Environment, Graduate School of Environment and Disaster Research, Tokoha University, Suruga, Japan; ^8^Center for Information and Neural Networks, National Institute of Information and Communications Technology, Suita, Japan

**Keywords:** mismatch negativity (MMN), prediction, electrocorticogram (ECoG), macaque, schizophrenia

## Abstract

Sensory prediction is considered an important element of mismatch negativity (MMN) whose reduction is well known in patients with schizophrenia. Omission MMN is a variant of the MMN which is elicited by the absence of a tone previously sequentially presented. Omission MMN can eliminate the effects of sound differences in typical oddball paradigms and affords the opportunity to identify prediction-related signals in the brain. Auditory predictions are thought to reflect bottom-up and top-down processing within hierarchically organized auditory areas. However, the communications between the various subregions of the auditory cortex and the prefrontal cortex that generate and communicate sensory prediction-related signals remain poorly understood. To explore how the frontal and temporal cortices communicate for the generation and propagation of such signals, we investigated the response in the omission paradigm using electrocorticogram (ECoG) electrodes implanted in the temporal, lateral prefrontal, and orbitofrontal cortices of macaque monkeys. We recorded ECoG data from three monkeys during the omission paradigm and examined the functional connectivity between the temporal and frontal cortices by calculating phase-locking values (PLVs). This revealed that theta- (4–8 Hz), alpha- (8–12 Hz), and low-beta- (12–25 Hz) band synchronization increased at tone onset between the higher auditory cortex and the frontal pole where an early omission response was observed in the event-related potential (ERP). These synchronizations were absent when the tone was omitted. Conversely, low-beta-band (12–25 Hz) oscillation then became stronger for tone omission than for tone presentation approximately 200 ms after tone onset. The results suggest that auditory input is propagated to the frontal pole *via* the higher auditory cortex and that a reciprocal network may be involved in the generation of auditory prediction and prediction error. As impairments of prediction may underlie MMN reduction in patients with schizophrenia, an aberrant hierarchical temporal-frontal network might be related to this pathological condition.

## Introduction

Our brain actively reconstructs the external world *via* predictions based on the learning patterns of sensory events. Such predictive coding is thought to be implemented within hierarchically organized neural circuits. Predictive signals are hypothesized to be sent *via* top-down connections, whereas prediction error signals derived from mismatches between predictions and actual sensory inputs are returned *via* bottom-up connections (1–4).

In the auditory system, the neural mechanisms involved in prediction and prediction error have commonly been studied using the mismatch negativity (MMN) framework. MMN is a negative deflection of the auditory event-related potential (ERP) elicited by an abrupt change in a sound stimulus after prior repetition of that sound (5). MMN reduction is one of the most robust observations in patients with schizophrenia (6, 7). Therefore, an understanding of the neural mechanisms underlying prediction and the prediction error of MMN is of particular interest.

Prediction occurs in the absence of the expected tone stimulus (8). Previous studies have shown that when sound stimuli are occasionally omitted after prior repetition of the same sound, they elicited MMN (9–11) or mismatch activities in MEG (12–14). As external input is lacking, a response to a predicted but omitted stimulus is considered a genuine prediction-related signal (PRS). Importantly, mismatch activities using the omission paradigm were reduced in patients with schizophrenia (14, 15). Therefore, PRSs may be related to the pathophysiology of schizophrenia (16).

Auditory MMN is localized to the temporal and frontal cortices. This has been confirmed in various ways, including source estimation in human (17, 18) and monkey (19, 20) electroencephalography (EEG) scans, and high-resolution electrocorticogram (ECoG) recordings from humans (21) and monkeys (22, 23). MacLean and Ward (18), using EEG source estimation, showed that phase coherence was apparent between the temporal and frontal cortices and may play a role in MMN generation (18).

Despite these works, we do not know how prediction signals are communicated between the temporal and frontal cortices, or how prediction errors are computed using predictive and sensory inputs. This is because it is difficult to quantify information propagation in most human recordings, and it is challenging to interpret any observed connectivity. Recent studies have found that feedforward and feedback connections were processed using distinct frequency band channels (24, 25). Thus, exploration of frequency band-dependent connectivities may reveal prediction-related, feedforward, and feedback information propagation between the temporal and frontal areas. In this study, we used ECoGs to record the detailed neural responses of the omission paradigm in macaque monkeys. Nonhuman primates have high homology with humans, especially in the structure of the frontal and auditory cortices, and showed comparative MMN in scalp EEG (7). Anatomical studies in monkeys have shown that the lateral belt of the auditory cortex (at the bank of the lateral sulcus) is reciprocally connected to multiple frontal cortical regions including the lateral prefrontal and orbitofrontal cortices; dual parallel pathways (the ventral and dorsal streams) were evident (26). We thus recorded data from the temporal cortex (including various subregions of the lateral belt) and the lateral prefrontal and orbitofrontal cortices and analyzed frequency band-dependent connectivities between the temporal and frontal regions.

## Methods

### Subjects and ECoG Implantation

**Figure 1:**
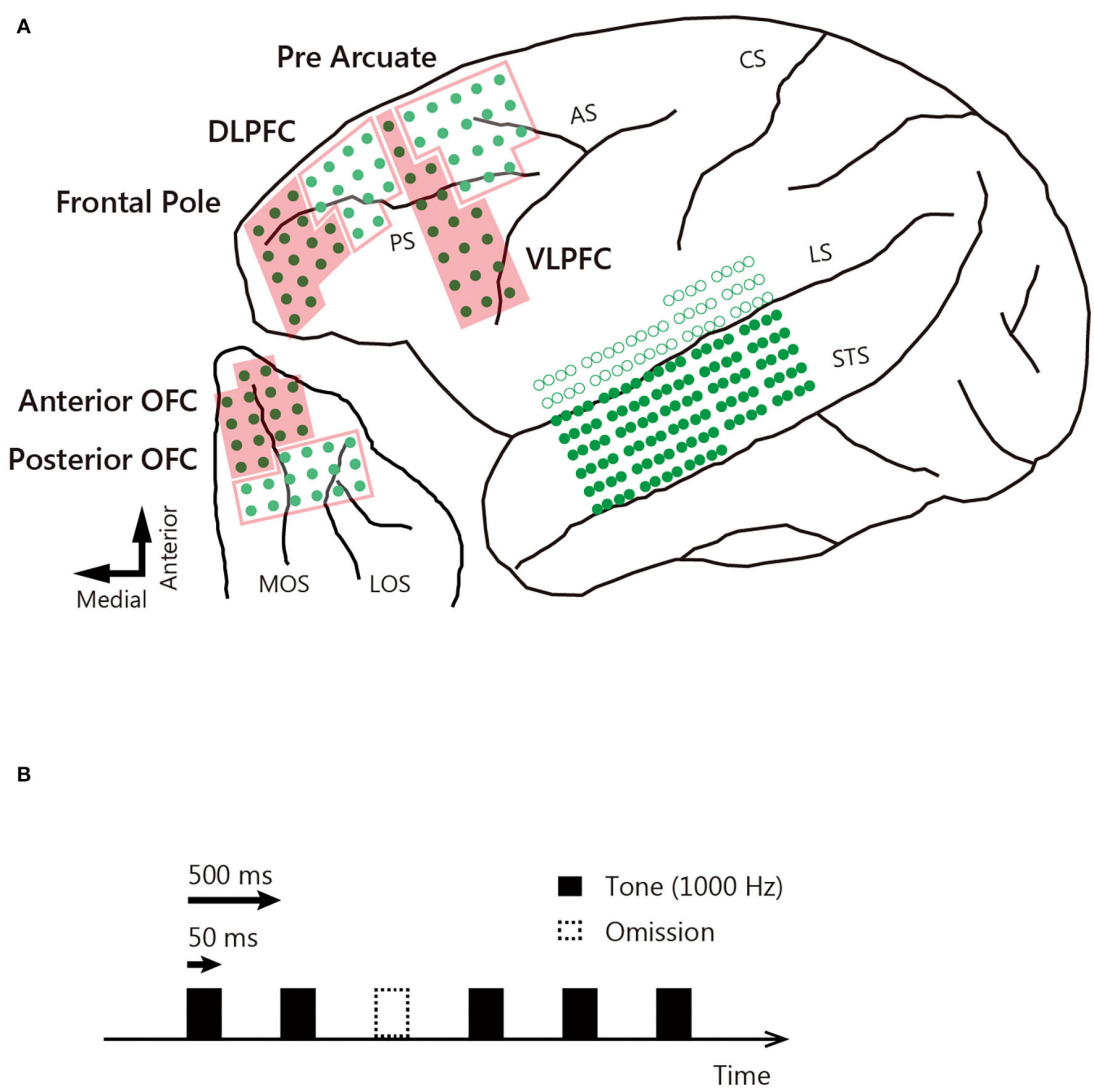
Locations of ECoG electrodes and auditory stimuli. **(A)** The ECoG electrodes covered the left hemisphere, and the exact locations were determined in monkey D on postmortem brain reconstruction. Green dots indicate electrodes located on the surface of the brain, and circles are electrodes inserted into the lateral sulcus. Arrows correspond to the direction of the lower left panel. STS, superior temporal sulcus; LS, lateral sulcus; CS, central sulcus; AS, arcuate sulcus; PS, principal sulcus; MOS, medial orbital sulcus; LOS, lateral orbital sulcus. **(B)** Auditory stimuli (1,000 Hz, 50 ms duration tone) were presented with a stimulus interval of 500 ms. Ten percent of the auditory stimuli were omitted. Black bars indicate tone presentation, and dotted bars indicate tone omission.

Three male Japanese macaques (*Macaca fuscata*) weighing 6.0 kg (monkey J), 5.3 kg (monkey D), and 4.9 kg (monkey N) were used. After training the monkeys to sit calmly in a dedicated chair, they were implanted with subdural ECoG electrode sheets in the temporal cortex (160 channels), lateral prefrontal cortex (64 channels), and orbitofrontal cortex (OFC: 32 channels) of the left hemisphere (Figure 1A) and with a head post (to allow head fixation). The work was performed at Niigata University using the standard aseptic surgical procedures described previously (27). The electrode grids were fabricated on 20 mm thick, flexible parylene-C film using microelectro-mechanical technology. All electrodes of the sheets placed in the lateral prefrontal cortex and OFC were 1.0 × 1.0 mm in dimension and spaced at 2.5 mm. The electrodes of the temporal cortex sheets were 0.3 × 0.3 mm in dimension and spaced at 1.0 mm on the anterior–posterior axis and 2.5 mm on the dorsal–ventral axis. For each temporal cortex, the sheet was inserted into the lateral sulcus and positioned on the superior temporal gyrus (Figure 1A). For each lateral prefrontal cortex and OFC, the sheet was positioned above the area. Reference electrodes were placed on the inner surface of the dura mater, over the parietal lobe. We used structural magnetic resonance imaging to confirm the correct placement of the implants. All animal care and experimental procedures were approved by Niigata University, Tamagawa University, and the University of Yamanashi Animal Care and Use Committee and were in accordance with the guidelines of the National Institutes of Health.

### Auditory Stimuli and Experimental Protocol

Experiments were conducted at Tamagawa University and the University of Yamanashi. Each monkey sat in a dedicated chair with the head fixed to face a 17-inch, thin-film transistor flat-screen monitor. To avoid body motion and sleep, the monkeys performed a fixation task during tone presentation and were rewarded with liquid when they fixated the center of the display for 2 s. A 1,000 Hz tone 50 ms in duration served as the auditory stimulus. This was presented every 500 ms, with a 10% probability of omission (Figure 1B). In each session, 900 tones were presented and 100 tones were omitted. The sound pressure was 80 dB, and the rise/fall times were 1 ms; the tones were presented binaurally through earphones. Data were collected over 10 sessions for each monkey. We used MATLAB (MathWorks) to control the behavioral tasks. Eye position was monitored using a remote eye-tracker system (iREC2).

### Electrophysiological Recordings and Data Analyses

We recorded ECoG data from 256 channels for each monkey during auditory stimulus presentation or omission. ECoG signals were filtered from 0.3 to 500 Hz and digitized at 1 kHz using dedicated hardware (Cerebus System, Blackrock Microsystems Inc.). We used EEGLAB software (28) for offline analyses. For each electrode, we calculated auditory ERPs elicited by tone presentation and omission. After bandpass (1–30 Hz) filtering, we extracted ECoG data from −200 to 600 ms after all event onsets of all trials. We rejected trial epochs exceeding ±1,000 μV at any electrode. Tone presentation trials were randomly selected to equate the number of trials to the number of tone omissions. ERPs associated with tone presentation and omission were calculated by averaging all trial responses across 16 electrodes in each subregion recorded in the 3 monkeys. We used the one-sample *t*-test to determine whether the ERP deviated from zero at each time point. The false discovery rate (FDR) was controlled using the Benjamini/Hochberg approach.

To evaluate functional connectivities between electrodes, we computed phase-locking values (PLVs). A PLV denotes the degree of phase difference consistency across trials and shows event-related phase coherence between two data time series (29). The PLV is defined as the average vector length calculated from phase angle differences as follows:


$$\begin{array}{l} \text{PLV}=\;|n^{-1}\sum_{t=1}^{n}e^{ip}| \end{array}$$

where *n* denotes the number of trials, *e* denotes the natural logarithm, *i* denotes the imaginary unit, and *p* denotes the phase angle difference in radians of each *t* trial. To avoid synchrony attributable to noise, we re-referenced all data by subtracting the mean response across each 32-channel connector from the raw response, followed by notch filtering (48–52 Hz) prior to calculating PLVs. We focused on theta- (4–8 Hz), alpha (8–12 Hz), low-beta- (12–25 Hz), high beta- (25–35 Hz), and gamma- (35–60 Hz) band PLVs.

We calculated PLVs between 2 temporal seed electrodes and 96 frontal electrodes (2 × 96 electrode pairs). For the anterior temporal cortex, we identified several electrodes that showed greater ERP response during tone omission than during tone presentation. Of these, the electrode located near the edge of the lateral sulcus (presumably corresponding to the anterior parts of the lateral belt) was selected as the seed electrode. For the posterior temporal cortex, the electrode placed near A1 (presumably ML) was selected as the seed electrode. We used the Circular Statistics Toolbox for Matlab to statistically test the significance of PLVs and the PLV difference between tone presentation and omission (30). To examine the significance of PLVs, we used a Rayleigh test to determine whether the distribution of phase angle difference deviated from uniformity (**Figures 3**, **4**). To examine the significance of the PLV difference between tone presentation and omission, we used a circular analog of the Kruskal-Wallis test (circ_cmtest) to determine whether the median phase angle between tone presentation and omission was significantly different for each frequency band (30). The PLV difference for each frequency band was determined to be significant if more than 20% of the data points within the frequency band were deemed significant (**Figure 5**). To summarize the areal difference and time course of PLV difference between tone presentation and omission, we calculated the proportion of significant electrodes within a 10 ms time window averaged across 16 channels for 3 representative areas (**Figure 6**). All statistical analyses were performed using custom scripts written in MATLAB R2014a (MathWorks).

## Results

### Tone Presentation and Omission Responses of the Temporal and Frontal Cortices

**Figure 2:**
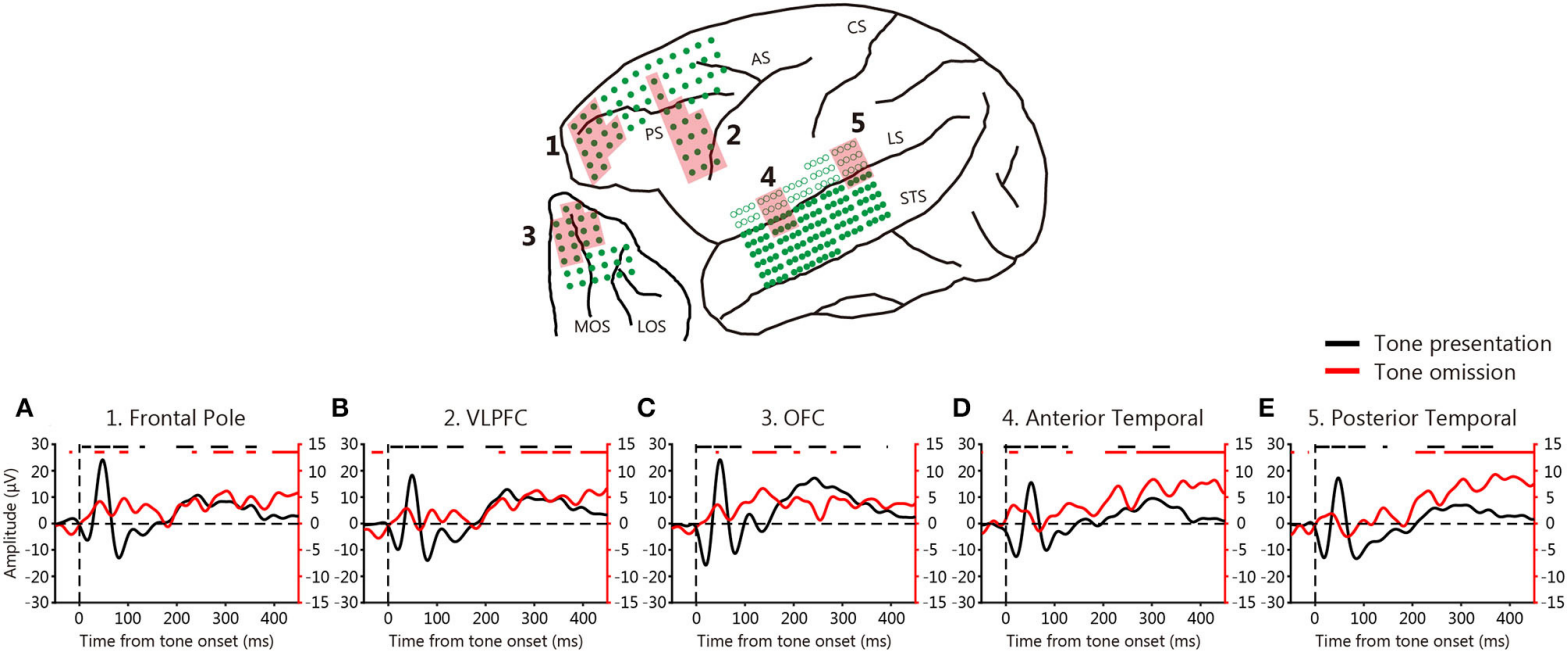
Event-related potentials (ERPs) during the omission paradigm. **(A–E)** ERPs averaged across 3 monkeys. Each line shows the average ERP across 16 electrodes in each area (red shading) that were averaged across 3 monkeys. Black lines: ERPs at tone presentation; red lines: ERPs at tone omission. The black and red lines on the tops of the graphs indicate deflections that deviated from zero (one-sample *t*-test, *p* < 10^−6^, FDR-corrected).

We first calculated ERPs associated with tone presentation and omission by averaging the responses to all event onsets. Figure 2 shows the ERPs to tone presentation (black) and omission (red) averaged across electrodes and monkeys in various areas of the temporal and frontal cortices. The ERPs to tone presentation occurred immediately after stimulus onset. The ERPs in the posterior temporal cortex were initially sharply negative (20 ms), and then turned positive (50 ms) and negative once more (90 ms), before exhibiting a positive deflection at around 200–300 ms (one-sample *t*-test, *p* < 10^−6^, FDR corrected). Similar patterns were observed for the frontal pole, ventro-lateral prefrontal cortex (VLPFC), OFC, and anterior temporal cortex in the three monkeys.

However, the ERPs to tone omission did not exhibit any sharp deflection, the timing of which varied among the cortical areas. An early (0–100 ms) positive deflection was first observed in the anterior temporal cortex, followed by the frontal pole and OFC (one-sample *t*-test, *p* < 10^−6^, FDR corrected). A late (from 200 ms) gradual increase was evident in the anterior and posterior temporal cortices (one-sample *t*-test, *p* < 10^−6^, FDR corrected), which was also slightly evident in the prefrontal cortices. The early positive deflection in the frontal pole was observed in all three monkeys, whereas it was observed in the anterior temporal cortex and OFC in two of the three monkeys (one-sample *t*-test, *p* < 0.0005, FDR-corrected). The late gradual increase was observed in all three monkeys (one-sample *t*-test, *p* < 0.005, FDR-corrected). These results suggest that the absence of a predicted auditory input elicits PRSs across a widespread network that includes both the temporal and frontal cortices. As an early positive deflection was observed, we hypothesized that PRSs might be generated in the anterior temporal cortex that is communicated to the frontal pole and OFC.

### Functional Connectivity Between the Auditory and Prefrontal Cortices During Tone Presentation and Omission

**Figure 3:**
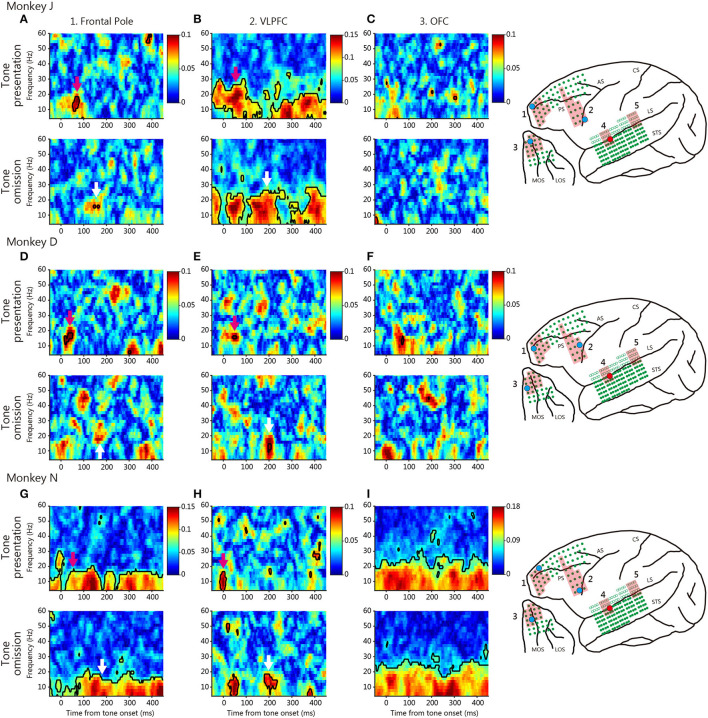
Typical phase-locking values (PLVs) between the anterior temporal cortex and various prefrontal cortical regions. The red dot on the brain map indicates the seed electrode, and the cyan dot indicates the target electrode. The red shaded regions indicate the defined area of the target electrode positioned. **(A–C)** PLVs of monkey J. The PLV map of each area is between the anterior temporal cortex electrode and the frontal pole **(A)**, the VLPFC **(B)**, and the OFC **(C)** electrodes, in terms of tone presentation (above) and tone omission (bottom). Black contoured lines indicate statistically significant PLVs (Rayleigh test, *p* < 0.05, FDR-corrected). Colored arrows indicate important patterns of PLVs in each tone. Color scales were identical for tone presentation and tone omission, but were different across area and monkey. **(D–F)** PLVs of monkey D. **(G–I)** PLVs of monkey N.

**Figure 4:**
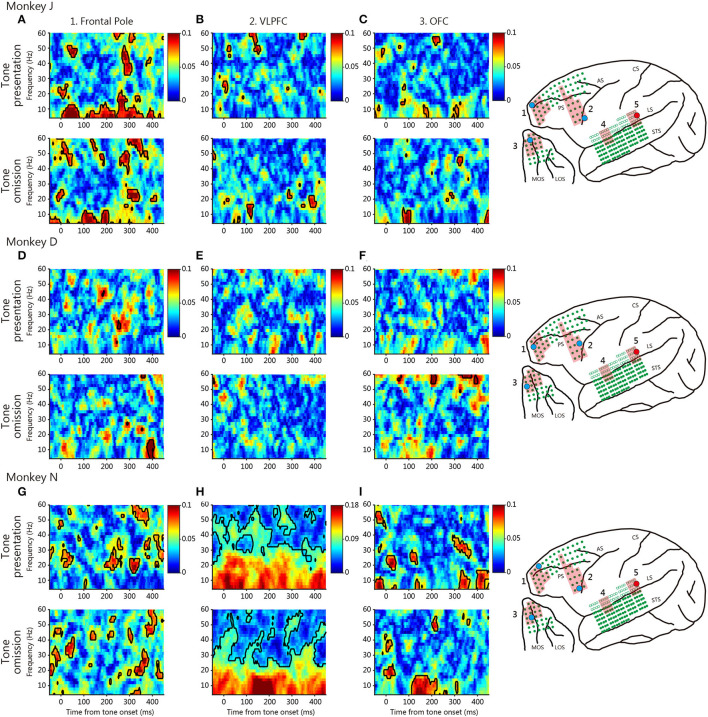
Typical phase-locking values (PLVs) between the posterior temporal cortex and various prefrontal cortical regions. The conventions are those of Figure 3.

To evaluate functional connectivity between the temporal and prefrontal cortices, we calculated the PLVs (Figures 3, 4). We evaluated the roles played by the anterior and posterior temporal cortices, because the auditory system features two parallel pathways (26). We identified seed electrodes within the anterior and posterior temporal cortices that elicited robust positive deflections during tone omission (Figure 2) and then calculated the PLVs between the temporal electrodes and various PFC areas. The seed electrodes lay in the lateral belt area (the AL or RTL for the anterior temporal cortex, and the ML for the posterior temporal cortex).

Figure 3 shows typical PLVs between the anterior temporal cortex and various prefrontal regions for each monkey. We found event-locked changes in the PLVs of different frequencies during both tone presentation and omission. At tone presentation, theta-low-beta frequency PLV was observed between the anterior auditory cortex and the frontal pole, the VLPFC; this developed about 100 ms after tone onset (magenta arrow). These results suggest that theta-low-beta frequency PLVs between these areas reflect the communication of the auditory input signal.

At tone omission, the frontal pole and the VLPFC theta-low-beta frequency PLVs developed about 200 ms after the predicted onset for the three monkeys (white arrow). The existence of theta-low-beta frequency PLV during omission suggests that it represents a communication relevant to the PRSs. No specific pattern was observed in the high-frequency range. For comparison, Figure 4 shows typical PLVs between the posterior temporal cortex and various prefrontal regions. No consistent between-monkey pattern is evident.

**Figure 5:**
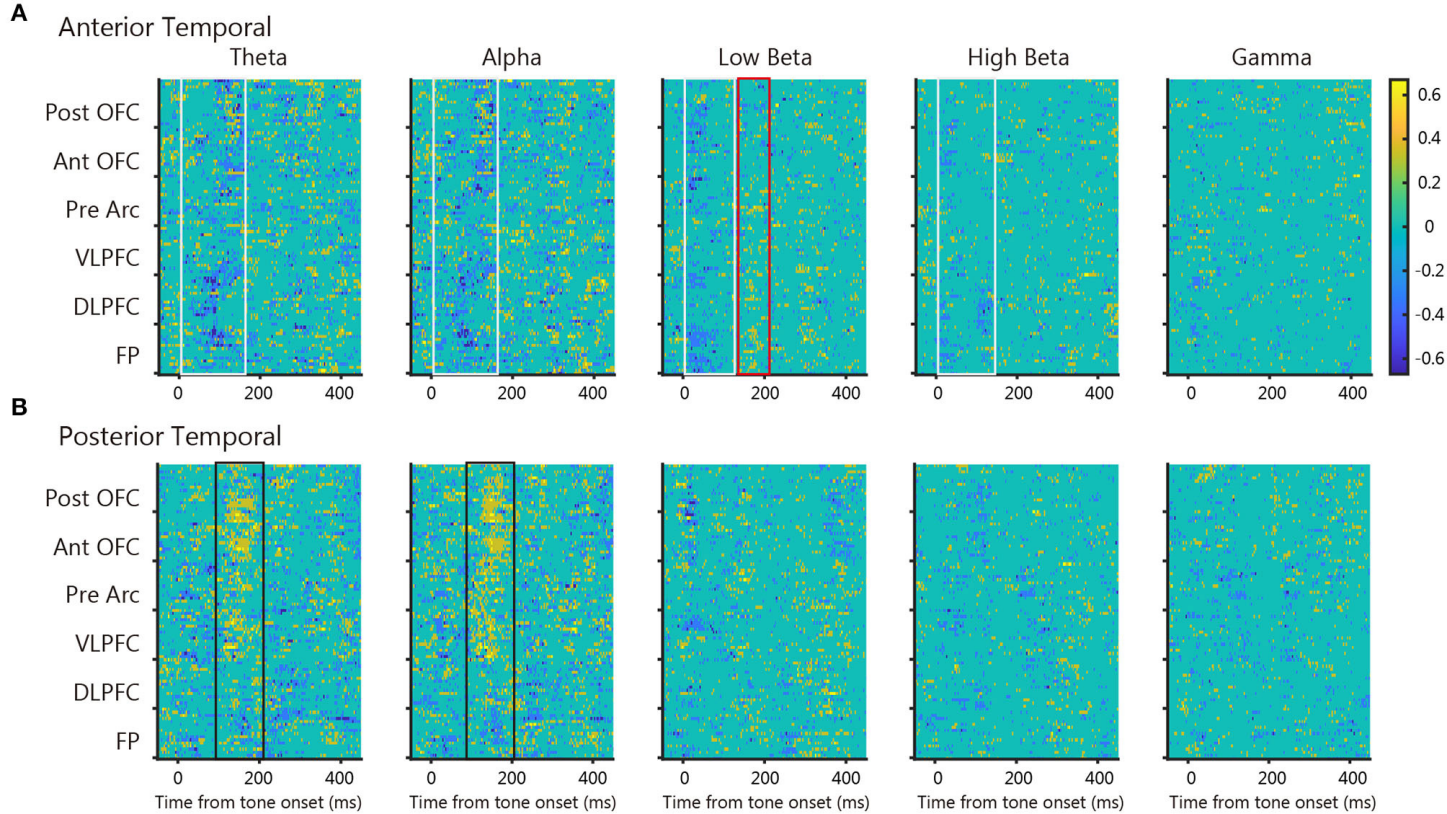
The statistical difference map between tone presentation and tone omission PLV. Each map shows the time course of significant difference ratio of the three monkeys between tone presentation and tone omission PLVs for all prefrontal electrodes at each frequency band (cmtest, *p* < 0.05, uncorrected). Blue indicates that the tone presentation PLV was significantly larger than the tone omission PLV, and yellow *vice versa*. Colored boxes indicate important patterns in each map. **(A)** Maps of the anterior temporal seed. **(B)** Maps of the posterior temporal seed.

Next, we quantified the frequency-dependent PLV differences between tone presentation and omission. Figure 5 shows the time course of significant difference ratio of the three monkeys between tone presentation and omission for all prefrontal electrodes; blue indicates that the tone presentation PLVs were significantly larger than the tone omission PLV, and yellow *vice versa*. For the anterior temporal seed, tone presentation PLVs were larger than tone omission in the theta- to high-beta range around 100 ms after tone onset (Figure 5A, blue, white box). Conversely, tone omission PLVs were larger than tone presentation in the low-beta band around 200 ms after tone onset (Figure 5A, yellow, red box). For the posterior temporal seed, tone omission PLVs were larger than tone presentation PLVs in the theta- and alpha-band around 150 ms after tone onset (Figure 5B, yellow, black box), whereas PLVs near tone onset were only modest. These results suggest that both the anterior and posterior temporal cortices were involved in the communication of PRS.

**Figure 6:**
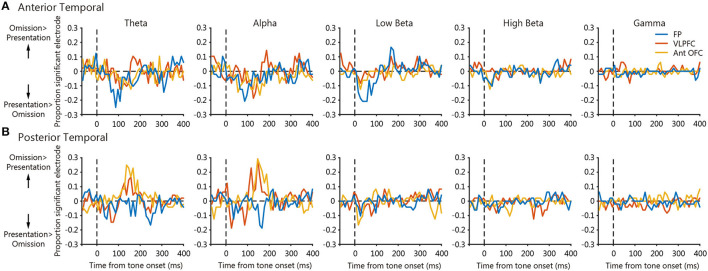
The time courses of the statistical difference map across prefrontal areas. Each panel shows the mean time course of proportion significant electrodes within 3 regions, namely, the frontal pole, the VLPFC, and the anterior OFC. Positive values indicate the proportion of significant electrodes where tone omission PLV was significantly larger than the tone presentation PLV, and negative values *vice versa*. **(A)** Panels of the anterior temporal seed. **(B)** Panels of the posterior temporal seed.

To determine the areal difference of the frequency-dependent PLVs between the temporal cortex and the prefrontal regions, we calculated the proportion of significant electrodes within three regions, namely, the frontal pole, the VLPFC, and the anterior OFC for each frequency band (Figure 6). Positive values indicate the proportion of electrodes with larger tone omission PLVs than tone presentation PLVs, and negative values *vice versa*. For the anterior temporal seed, tone presentation PLVs were larger than tone omission PLVs around 50–100 ms in the theta-, alpha-, and low-beta-bands for the frontal pole electrodes (Figure 6A, blue). This was followed by larger tone omission PLVs around 150–200 ms after tone onset in the theta- and alpha-bands for the VLPFC electrodes (Figure 6A, red) and in the low-beta-band for the frontal pole electrodes (Figure 6A, blue). For the posterior temporal seed, tone omission PLVs were larger than tone presentation PLVs around 100–200 ms after tone onset in the theta- and alpha-bands for the VLPFC and the anterior OFC electrodes. Overall, tone presentation-specific synchronization was mainly observed between the anterior temporal cortex and the frontal pole, and tone omission-specific synchronization was apparent between the anterior temporal cortex and the frontal pole, the VLPFC, and between the posterior temporal cortex and the VLPFC, OFC.

## Discussion

We found that PRSs were widespread in the temporal and frontal cortices. Because the early-latency ERP for tone omission arose earlier for the temporal cortex compared with the frontal pole and OFC, PRSs were presumably generated in the temporal cortex and propagated to the frontal pole and OFC. However, it was difficult to determine how they were propagated: we did not find early phase synchrony for tone omission. Beta-band synchronization was observed between the anterior temporal cortex and the frontal pole around 150 ms after the absence of auditory stimulation, suggesting a late communication of PRSs. Conversely, the VLPFC and the OFC were synchronized in the theta- and alpha-band range between the frontal cortices and the posterior temporal cortex. These synchronizations may be related to the generation of the gradual increase in ERP after 200 ms observed in the temporal cortex, which we interpret as a PRS, although it may merely reflect anticipatory effects. Signals relevant to auditory input were communicated reciprocally between the anterior auditory cortex and the frontal pole. These areas became synchronized in the theta-, alpha-, and low-beta-band range around 80 ms after tone onset.

Previous human studies that examined responses when the omission paradigm was in play reported that omission responses developed in the temporal and frontal areas (12, 13). Our results in the macaque monkey are in line with these studies. The omission responses recorded in temporal areas in previous studies were modulated by the attention paid to tone stimuli (13, 31), which implies that a top-down signal from prefrontal areas contributed to the derivation of the omission response in the temporal area. Furthermore, dipole sources (identified using MEG) (13) and intracranial recordings of omission responses (31) were localized to the posterolateral frontal cortex, suggesting that the connection between the lateral prefrontal and temporal cortices is critical in terms of eliciting responses to omission. In this study, we directly showed that responses to an omission in the lateral prefrontal and temporal cortices were phase-synchronized. As the synchrony lay in the beta-band range, we speculate that it reflects top-down propagation of PRSs (24, 32), although another study suggested that beta-band synchronization reflects bottom-up propagation during the resting state (25).

To this point, we have described omission responses as PRSs. Such signals feature (at least) prediction and prediction error. The omission paradigm allowed us the possibility to distinguish the two because it eliminates the effects of sound differences in typical oddball paradigms. In general terms, responses to only tone presentation may principally be sensory inputs, those to both tone presentation and omission may be predictions, and those to only tone omission may be prediction errors. Gradual positive deflections in the ERPs were observed at both tone presentation and omission and are considered to contain prediction signals. Conversely, the beta-band PLV between the anterior auditory cortex and the frontal pole was larger at tone omission compared with presentation and is considered to contain prediction error signals. In reality, it is difficult to conclude that a response is attributable to one condition and not another. For example, responses to both tone presentation and omission may be caused by sensory inputs and prediction error instead of predictions. This is particularly true for heterogeneous signals, such as those observed in ECoG and EEG. Thus, although the omission paradigm renders it possible to distinguish prediction from prediction error signals, signal assignments are necessarily tentative. Indeed, only a few studies have successfully isolated prediction signals. Ohmae et al. (8) found prediction signals in the cerebellar nucleus using the omission paradigm. It was suggested that responses in the cerebellar nucleus corresponded to prediction signals because the responses increased with successive tone presentation and became maximal at tone omission. Future studies examining whether the ERP responses increased with successive tone presentations are warranted.

In this study, we aimed to obtain prediction-related signals that may be related to neuropsychiatric disorder (16). Therefore, we utilized a long ISI (500 ms) oddball paradigm that is typically used in clinical studies. However, a previous human scalp EEG study that examined responses in the omission paradigm reported that only a short ISI (below 150 ms) elicited a clear negative deflection in a fronto-central site (33). Conversely, a previous MEG study found that longer ISI (1,000 ms) elicited a mismatch response in the omission paradigm that was reduced in patients with schizophrenia (14). In addition, a previous macaque study reported that prediction does occur for longer ISIs (8). Therefore, prediction signals for longer ISIs may not be detectable in EEG experiments, although they exist in the brain, and more complex paradigms may be needed to detect omission MMN in scalp EEG recording (11).

In conclusion, we found communication of PRSs between the anterior temporal cortex and the frontal pole in the omission paradigm, a variant of the typical oddball MMN paradigm. These results may not necessarily extrapolate to humans and thus patients with schizophrenia. However, auditory prediction is considered as one of the important elements of MMN whose reduction is well known in patients with schizophrenia (7). Furthermore, patients with schizophrenia are possibly impaired in their predictive abilities, demonstrated both behaviorally (34) and by non-invasive means, including impairments in the omission paradigm (14, 15, 35). Thus, an understanding of the neural mechanisms underlying the communication of PRSs may enhance our knowledge of the neural impairments underlying schizophrenia.

## Data Availability Statement

The raw data supporting the conclusions of this article will be made available by the authors, without undue reservation.

## Ethics Statement

The animal study was reviewed and approved by Animal Care and Use Committee: Niigata University, Tamagawa University, and University of Yamanashi.

## Author Contributions

TSU constructed the electrodes. TMA, KKAW, and IH performed the surgery. YS, TSA, MI, TMI, and HK collected the data. YS, MT, KKI, and TU analyzed the data. YS, MT, and TU interpreted the results and wrote the manuscript.YS, MT, KM, KKAS, and TU designed the study. KM, IH, KKAS, and TU supervised all aspects of data collection, analysis, and interpretation. All authors commented on the manuscript and contributed to and approved the final manuscript.

## Funding

This study was supported by a Brain Mapping by Integrated Neurotechnologies for Disease Studies (Brain/MINDS) grant from the Japan Agency for Medical Research and Development (AMED) (No. 15653128 to KKAS and KM and JP19dm0207069 to KKAS and TU).

## Conflict of Interest

The authors declare that the research was conducted in the absence of any commercial or financial relationships that could be construed as a potential conflict of interest.

## Publisher's Note

All claims expressed in this article are solely those of the authors and do not necessarily represent those of their affiliated organizations, or those of the publisher, the editors and the reviewers. Any product that may be evaluated in this article, or claim that may be made by its manufacturer, is not guaranteed or endorsed by the publisher.
